# PET/CT and SPECT/CT Imaging of HER2-Positive Breast Cancer

**DOI:** 10.3390/jcm12154882

**Published:** 2023-07-25

**Authors:** Jeremy McGale, Sakshi Khurana, Alice Huang, Tina Roa, Randy Yeh, Dorsa Shirini, Parth Doshi, Abanoub Nakhla, Maria Bebawy, David Khalil, Andrew Lotfalla, Hayley Higgins, Amit Gulati, Antoine Girard, Francois-Clement Bidard, Laurence Champion, Phuong Duong, Laurent Dercle, Romain-David Seban

**Affiliations:** 1 Department of Radiology, Columbia University Medical Center, New York, NY 10032, USA; 2 Molecular Imaging and Therapy Service, Memorial Sloan Kettering Cancer Center, New York, NY 10065, USA; 3 School of Medicine, Shahid Beheshti University of Medical Sciences, Tehran 1985717443, Iran; 4 Campbell University School of Osteopathic Medicine, Lillington, NC 27546, USA; 5 American University of the Caribbean School of Medicine, Cupecoy, Sint Maarten; 6 Touro College of Osteopathic Medicine, Middletown, NY 10940, USA; 7 Department of Internal Medicine, Maimonides Medical Center, New York, NY 11219, USA; 8 Department of Nuclear Medicine, CHU Amiens-Picardie, 80054 Amiens, France; 9 Department of Medical Oncology, Inserm CIC-BT 1428, Curie Institute, Paris Saclay University, UVSQ, 78035 Paris, France; 10 Department of Nuclear Medicine and Endocrine Oncology, Institut Curie, 92210 Saint-Cloud, France; 11 Laboratory of Translational Imaging in Oncology, Paris Sciences et Lettres (PSL) Research University, Institut Curie, 91401 Orsay, France

**Keywords:** HER2, breast cancer, oncology, PET/CT, PET, SPECT/CT, SPECT medical imaging

## Abstract

HER2 (Human Epidermal Growth Factor Receptor 2)-positive breast cancer is characterized by amplification of the HER2 gene and is associated with more aggressive tumor growth, increased risk of metastasis, and poorer prognosis when compared to other subtypes of breast cancer. HER2 expression is therefore a critical tumor feature that can be used to diagnose and treat breast cancer. Moving forward, advances in HER2 in vivo imaging, involving the use of techniques such as positron emission tomography (PET) and single-photon emission computed tomography (SPECT), may allow for a greater role for HER2 status in guiding the management of breast cancer patients. This will apply both to patients who are HER2-positive and those who have limited-to-minimal immunohistochemical HER2 expression (HER2-low), with imaging ultimately helping clinicians determine the size and location of tumors. Additionally, PET and SPECT could help evaluate effectiveness of HER2-targeted therapies, such as trastuzumab or pertuzumab for HER2-positive cancers, and specially modified antibody drug conjugates (ADC), such as trastuzumab-deruxtecan, for HER2-low variants. This review will explore the current and future role of HER2 imaging in personalizing the care of patients diagnosed with breast cancer.

## 1. Introduction

Breast cancer is the most commonly diagnosed cancer in women and the eighth leading cause of female mortality worldwide [1,2]. Screening for breast cancer typically begins around age 50 (per guidelines such as the United States Preventative Service Task Force (USPSTF)) and can help identify early-stage disease as well as decrease delays in treatment initiation, ultimately leading to better outcomes. Once a breast cancer is identified, it can be further characterized by its underlying mutations. Of particular interest are mutations involving the HER2 gene, which encodes a membrane tyrosine kinase in the epidermal growth factor (EGFR) family of receptors that are essential for epithelial cell growth, differentiation, and angiogenesis [3]. Pathologic amplification of this oncogene results in HER2 receptor overexpression and is a major driver of tumor development and progression in about 15% of all breast cancers [3,4]. Detailed characterization of HER2 status in tumors allows for the deployment of highly-targeted and effective treatments. Herein, we first review these treatments before describing how HER2 status has traditionally been determined and how imaging is being employed as an alternative diagnostic strategy. Then, we detail recent research in specific radiotracers for improved HER2 quantification before looking into combined diagnostic/therapeutic modalities, exploring the applications of HER2 imaging beyond diagnostics (e.g., staging, prognostication, response assessment, and surveillance), and, finally, looking into new areas of active exploration in the field as well as identifying current gaps in knowledge.

## 2. Overview: HER2 Diagnostics and Therapeutics

Identification, at baseline and throughout treatment, of overall HER2 surface presence and distribution in a patient’s cancer could help clinicians make decisions about treatment dosing, length, and an eventual switch in primary agent if the need arises.

### 2.1. HER2 Targeted Therapy

Development of HER2-targeted therapies using monoclonal antibodies (e.g., pertuzumab and trastuzumab) and tyrosine kinase inhibitors (including tucatinib, which has been found to be superior to the previously used lapatinib) over the last decade has led to significant improvement in the survival of patients with known HER2-positive breast cancer. The former, antibody-based treatments target the extracellular portion of the HER2 receptor and are currently used in almost all lines of treatment, except for those regimens that only employ tyrosine kinase inhibitors. By antagonistically binding at the cell surface, they act to both decrease HER2-initiated cellular signaling (causing arrest in cell division) and increase immune-mediated cytotoxicity as the patient’s own defenses recognize antibody opsonization. However, despite the development of specific agents, HER2-targeted therapy still remains challenging, with a significant number of patients not responding or eventually becoming resistant to currently available therapies. To address this resistance, several new molecular entities have begun to enter the literature, including antibody-based fragments (Fab), diabodies, minibodies, nanobodies, affibodies, and various other monoclonal antibodies (mAb) [5]. Of particular note, a trastuzumab antibody conjugate (trastuzumab-deruxtecan, T-DXd), was recently approved by the FDA for treatment of HER2-low metastatic breast cancer after demonstrating superior outcomes over chemotherapy in the DESTINY-Breast04 trial [6].

### 2.2. HER2 In Vitro Diagnostic Testing

Due to the utility of mutated HER2 cancer as a therapeutic target, accurate characterization of tumors is paramount. The current standard-of-care for HER2 status determination includes immunohistochemistry (IHC) and fluorescence in situ hybridization (FISH) with an average sensitivity of 75.4% (range of 47% and 100%) and false negative rate of 24.6% [7,8]. IHC measures the expression of HER2 receptors on the cellular surface of a biopsied tissue sample, which can be graded as 0, 1+, 2+, or 3+, with 0 and 1+ classified as HER2-negative, 2+ classified as borderline/equivocal/HER2-low, and 3+ classified as HER2-positive [7]. For borderline/equivocal results, FISH analysis can describe amplification of the HER2 gene by direct visualization. Cancers that are HER2-positive, as determined by IHC and FISH, are the most aggressive and have historically been approached with HER2-targeted medications. However, it is important to note that current IHC assays were developed in order to detect HER2-positive tumors, not to distinguish between HER2-low and HER2-negative tumors, thus confounding specific characterization of the latter two. Given that HER2-low tumors can now be targeted with novel approved therapies, there is an urgent need to refine methodology in order to accurately detect and characterize tumors with low levels of HER2 expression [6].

**Figure 1:**
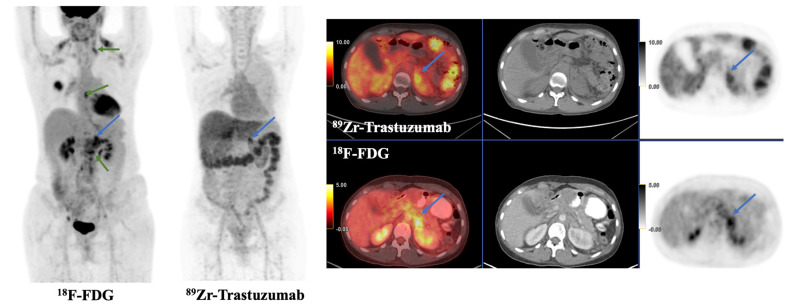
This is a 43-year-old female with left breast invasive ductal carcinoma (ER+, PR+, and HER2−) and metastases to the left supraclavicular, thoracic, and retroperitoneal nodes and left adrenal gland. She was on everolimus and exemestane at the time of ^89^Zr-trastuzumab PET/CT. Tracer avidity was visualized in a known left adrenal metastasis (blue arrow), with SUVmax 9.2. Additional FDG-avid left supraclavicular, thoracic, and retroperitoneal nodes were not tracer avid on ^89^Zr-trastuzumab PET/CT (green arrows), suggesting lack of HER2 expression and HER2 intertumoral heterogeneity of metastatic lesions.

In addition to the challenge of differentiating HER2-low tumors, in vitro diagnostic testing is limited by multiple dynamic factors, including intratumoral heterogeneity, the fact that HER2 expression can change over time, and the possibility that HER2 expression at the primary cancer biopsy site can be different when compared to that of a metastatic lesion (with discordance rates from 3% to 16%) [9,10]. This latter example is demonstrated by the case study in Figure 1. Ultimately, biochemical tests used to identify HER2 status are limited by the spatial and temporal heterogeneity of HER2 expression, an idea that is addressed with more systematic HER2 imaging.

### 2.3. HER2 In Vivo Diagnostic Testing with Imaging

^18^F-FDG PET/CT is already a nearly ubiquitous method for whole-body tumor diagnosis and in-treatment monitoring in breast cancer due to its high sensitivity and specificity for the detection of metastatic disease [11]. This broad scope tracer demonstrates variable uptake (or avidity) in different cancers according to individual glucose distribution and metabolism. Certain subtypes of breast cancer, such as invasive ductal carcinomas, show increased uptake in comparison to noninvasive ductal carcinomas, while even large sized lobular breast cancers may remain occult due to decreased ^18^F-FDG uptake [12,13]. FDG avidity of breast cancer also widely varies depending on receptor status, with ER-negative tumors showing higher radiotracer activity than ER-positive tumors [14].

However, while ^18^F-FDG PET/CT is generally effective for actively metabolizing entities, it is not a tumor specific tracer. Benign conditions associated with inflammation or infection can cause false-positives relating to tracer uptake, which, in turn, could lead to unnecessary or excess treatment [1]. By employing a strategy parallel to that described above for treatment, PET/CT and SPECT/CT (a technique that allows for the precise description of tissue perfusion and functionality) can be augmented for targeted detection of HER2-positive breast cancer. Receptor-specific SPECT- or PET-intense radiotracers allow for the localization and quantification of tumor HER2 surface protein expression.

## 3. HER2-Specific Imaging

Improving the specificity of radiotracer targeting could help with the optimization of treatment and prediction of overall therapeutic response. The HER2 receptor represents a critical radiolabeling target of interest, and several approaches have been developed for characterizing HER2-positive primary tumors and distant metastases. A summary of studies on the in-human use of HER2-specific radiotracers, collected via database searches of terms relating to PET/SPECT, HER2, and targeted radiotracers, is displayed in Table 1.

### 3.1. Monoclonal Antibodies

One of the more basic approaches for targeting HER2 receptors is by co-opting the existing, known, specific monoclonal antibody trastuzumab and combining it with a radiotracer detectable by PET imaging. A current formulation includes labeling with a desferrioxamine-chelated (DFO) zirconium ion, which, with optimal dosage and timing, allows for accurate visualization and quantification of HER2-expressing tumors by PET scan 4–5 days after tracer administration [17]. Additionally, in several patients with HER2-negative breast cancers, unexpected metastases were detected with ^89^Zr-DFO-trastuzumab [18]. This tracer is also being investigated for response assessment purposes, with several PET imaging studies including the IMPACT trial (IMaging PAtients for Cancer Drug SelecTion) at the University Medical Center Groningen, utilizing ^89^Zr-DFO-trastuzumab for baseline scans in order to predict eventual therapeutic response [14]. These are promising results despite the relatively short half-life of ^89^Zr (~78 h), which generally serves to make timed detection particularly challenging [46].

An alternate tracer utilizes radioactive copper with a 1,4,7,10-tetraazacyclododecane-1,4,7,10-tetraacetic acid (DOTA) chelate, similarly affixed to a trastuzumab monoclonal antibody core. Studies of this ^64^Cu-DOTA-trastuzumab label show that it has comparable radiation exposure to ^18^F-FDG PET and is capable of assisting with detection of both HER2-positive primary and metastatic breast cancer [32]. Additionally, ^64^Cu-DOTA-trastuzumab enhanced PET can efficiently diagnose brain metastases, thereby suggesting free passage across the blood brain barrier [35]. Further support for the precision and utility of this tracer are its high tumor-to-background and non-cardiotoxic cardiac uptake [14,47].

**Figure 2:**
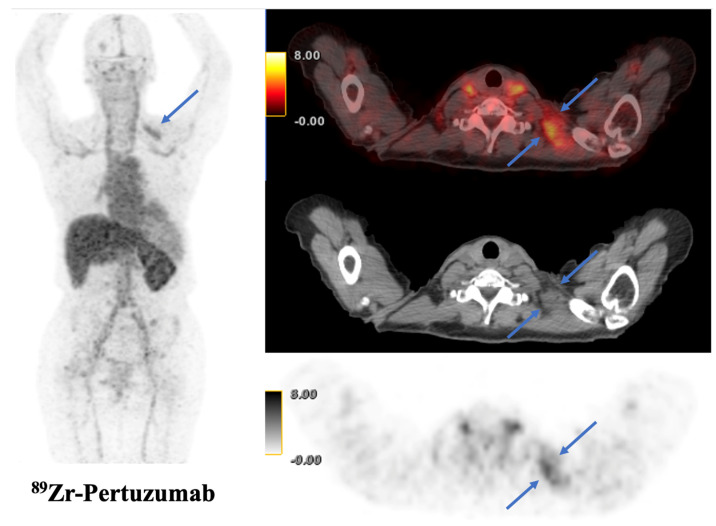
This is a 69-year-old female with left breast invasive ductal carcinoma (ER+, PR+, and HER2+) who has received several rounds of HER2-targeted therapy, including ado-trastuzumab emtansine (TDM-1), and was on gemcitabine, trastuzumab, and pertuzumab therapy at the time of ^89^Zr-pertuzumab PET/CT. Tracer avidity was visualized in a known left supraclavicular lymph node metastasis (blue arrows), with SUVmax 5.1.

Trastuzumab, while supplying an important base for many burgeoning radiolabels, has been implicated in the occasional detection of false positives. In response, pertuzumab, an antibody that binds to a distinct HER2 site, is being investigated with ^89^Zr based radiotracers [25,48]. A case example of a pertuzumab containing tracer is illustrated in Figure 2.

### 3.2. Affibodies and Nanobodies

Molecular imaging using affibodies is a new development in detecting and predicting HER2 status in breast cancers. Affibodies are small molecules approximately 6.5 kDa in size that are based on an immunoglobulin scaffold. ^111^Indium-ABY-002 and ^68^Gallium-ABY-002 are two examples that have been shown to have antibody-binding properties with fast pharmacokinetics. These were first studied in 2010, when Baum and colleagues utilized them for imaging HER2-positive breast cancer [43]. Patients with known metastases received radiolabeled affibodies, and good quality SPECT/CT and PET/CT images were obtained 2 h after injection. Most metastatic lesions identified on ^18^F-FDG PET were also seen on ABY-002 imaging, but lesions near the kidney and liver were not identified due to high background accumulation. An additional study by Sörensen and colleagues looked at a reengineered affibody, ABY-025, which had better tumor/background ratios and overall improved tumor uptake properties, helping to characterize liver metastasis [45].

Nanobodies, on the other hand, are “miniaturized” variants of monoclonal antibodies containing only two heavy chains with a single antigen-binding variable region. These entities retain antibody-binding properties but are highly modular and easily manipulated without requiring extensive assembly or technical optimization [49]. Keyaerts et. al. assessed safety, dosimetry and biodistribution of a ^68^Gallium based nanobody, showing that it had low background uptake and a favorable activity profile for the delineation of primary and metastatic disease [28]. Xavier et. al. reengineered a guanosine-phosphate HER2-nanobody, labeling it with ^18^F, and showed rapid clearance from the kidneys. This nanobody, when coadministered with trastuzumab, was also used to monitor therapeutic response [50].

### 3.3. SPECT/CT Imaging

The integration of SPECT and CT into a hybrid system brings new advantages for the diagnosis and treatment of breast cancer. CT provides comprehensive anatomic imaging while SPECT focuses on relaying information on tumor perfusion. Combined, SPECT/CT can be diagnostic by fusing morphologic and functional information, for example by detecting the precise locations of tumor metastases and mapping lymphatic drainage. The latter point specifically, and the resulting ability to help precisely localize lymph nodes, is an emerging application of SPECT/CT as sentinel lymph node dissection replaces axillary lymph node dissection for initial breast cancer staging [51]. Diagnostically, SPECT/CT has been shown to be as accurate as PET/CT while also providing the advantage of being more widely available and less costly [51].

SPECT/CT has a significant potential role in the future of HER2-targeted imaging. Targeted molecules radiolabeled with short half-life isotopes may be beneficial for patients due to faster HER2-positive tumor uptake and tissue clearance, decreasing overall radiation burden. SPECT/CT allows clinicians to work within the short half-lives of these tracers, such as those containing ^111^In and ^99m^Tc, as these images can be acquired more quickly after tracer administration than with PET/CT [52]. In one study, imaging using ^99m^Tc-HYNIC-H6F (with H6F being a HER2-specific peptide) was proven to detect HER2-positive tumors during trastuzumab therapy while not interrupting or blocking treatment administration [53]. SPECT/CT was able to visualize a significant amount of tracer uptake and detect HER2-positive tumors within 30 min of injection. The marker’s specificity also allowed it to quickly clear HER2-negative tissues, reducing unnecessary radiation applied to normal tissue.

## 4. HER2 Radionucleotide Therapy

Radiopharmaceuticals used to target HER2-positive cells can be used for both diagnostic and therapeutic purposes depending on the type of radionuclide attached to the biomarker or tracer. A radionuclide that emits tissue-penetrating gamma radiation is useful for imaging, whereas one that releases cytotoxic alpha, beta, or auger radiation can be used to eliminate tumor cells in the vicinity of the tracer. This forms the basis of radionuclide therapy, a novel treatment modality that can deliver systemic radiation directly and specifically to tumor cells. A radionuclide that can emit a detectable level of both kinds of radiation (gamma and cytotoxic) has great theragnostic value as it can be used to simultaneously treat as well as monitor response [54].

Targeted systemic radiation therapy requires an effective vehicle that can selectively deliver radionuclide to tumor cells alone and remain stable in the presence of emitted radiation. Trastuzumab has been the foremost tracer used to develop several radiotherapeutic agents in the past decade, as described above, and has been utilized in conjunction with a number of radioactive markers, such as ^131^I, ^177^Lu, ^64^Cu, ^111^In, and ^90^Y [55].

Among the radionuclides used to target HER2, beta-emitters have gained favor as a result of their short-range cytotoxic effects and ease of imaging due to concurrent gamma emission, a feature that could be useful in calculating overall radiation dose delivered. ^131^I specifically is widely used in therapy because of its cost-effectiveness, ease of manipulation and combination with antibodies, and reasonable half-life (t1/2 = 8.1 days) [56]. ^177^Lu, another beta-emitting isotope, can similarly be produced on a large scale at a low cost. It was shown to be cytotoxic in vitro on a breast cancer cell line while maintaining immunoreactivity with trastuzumab [57]. Alpha and Auger-emitters, such as ^111^In, might be better adapted for the treatment of small tumor foci due to shorter path length in the range of the cell’s diameter. A nuclear localizing signal (NLS) could also be used to efficiently deliver this tracer into tumor nuclei, thereby limiting toxicity to adjacent non-tumor cells [58].

Radionuclide therapy confers many benefits when compared to treatment with trastuzumab alone, including the ability to monitor dosage and progress, while potentially decreasing the required effective dosage of trastuzumab [59]. Research in the development of new radiopharmaceuticals is promising, and these preclinical studies warrant further investigation in clinical trials before becoming a part of the standard treatment regimen.

## 5. HER2 Imaging for Staging and Prognostication

Traditional breast cancer staging is based on the TNM classification system per the American Joint Committee on Cancer (AJCC) Staging Manual, which includes the primary tumor size (T), regional lymph node involvement (N), and distant metastasis (M) [60]. A combination of these factors determines the overall anatomic stage from 0 to IV, helping to guide treatment decisions. The 8th edition of this manual introduced additional characteristics to incorporate when determining stage, including tumor grade, biomarker status (ER, PR and HER2), and genomic panels. This update followed publication of data demonstrating superior prognostication ability of biomarker inclusion over TNM staging alone [61,62]. Though HER2 status has traditionally been quantified by IHC into broad categories primarily focusing on the distinction of high vs. “other” HER2 expression (including variants with low or no expression), more specific quantification may now be performed through targeted imaging [63].

Treatment resistance in HER2-positive breast cancers remains an area of active investigation. It is thought to result, at least in part, from failure to identify occult cancer foci on initial staging with diagnostic imaging, leading to inaccurate assessment of disease, prognostication, and associated treatment planning [64,65]. Certain tissues present diagnostic challenges to clinicians; bone, for example, is a common site for breast cancer metastasis, but is not well assessed for HER2 receptor status via IHC [66]. HER2 molecular imaging offers a non-invasive way to assess various foci of disease, as opposed to a single tissue which is traditionally obtained via biopsy. In their pilot study of 14 patients, Djikers et. al. found exceptional anti-HER2 tracer uptake in almost all known metastatic sites and even uncovered new foci of metastatic disease on delayed imaging [17].

## 6. HER2 Imaging for Response Assessment

There is currently a debate on the role for HER2 targeted PET/SPECT tracers in response assessment and an existing paucity of literature on the subject (outside of the ongoing and previously mentioned IMPACT trial). In theory, because HER2 tracers inherently only give information about surface expression of receptors, a decline in tracer uptake on serial scans would not necessarily indicate response to treatment; such a decline could just as easily mean that the tumor has mutated and lost HER2 expression due to tumor heterogeneity. Compared to ^18^F-FDG PET, which measures metabolic activity in the entire tumor regardless of surface expression or mutation, HER2-targeted tracers do not provide sufficient information for response assessment purposes. Perhaps the role is presently confined instead to assessing variable sensitivity to therapeutic agents (e.g., trastuzumab) throughout a treatment course.

## 7. HER2 Imaging for Surveillance

Similar to response assessment, the role of HER2-targeted PET/SPECT tracers in surveillance of breast cancer patients remains undetermined and needs to be evaluated further. By targeting HER2, PET and SPECT imaging could provide a non-invasive means to detect disease recurrence of HER2-positive tumors, offering higher sensitivity and specificity than traditional imaging modalities, including ^18^F-FDG PET/CT. Integration into routine surveillance protocols should be considered given its potential to improve breast cancer patient outcomes by enabling timely interventions and optimization of therapeutic strategy.

## 8. Future Directions

### 8.1. HER2-Low Cancers

Previously, only HER2-positive breast cancer could be targeted with anti-HER2 therapies, while HER2-zero and HER2-low patients were excluded from specialized treatment. Trastuzumab-deruxtecan, however, showed significant benefits compared to standard chemotherapy for treatment of patients with HER2-low metastatic breast cancer, prolonging both progression-free survival and overall survival [6]. These findings are leading to changes in the classification of advanced breast cancer, and methods for assessing HER2 status are actively being revised to accurately identify HER2-low patients [67,68].

The development of highly specific radiotracers is crucial in this endeavor as it may allow for more granular visualization of lesions with low HER2 expression, making patients previously classified as HER2-zero now eligible for T-DXd. It could also become a predictive marker of T-DXd efficacy and help identify patients who will not benefit from the treatment.

### 8.2. Artificial Intelligence and Radiomics

Radiomics is defined as extracting hidden parameters in the pixels of medical images (including MRI, CT, PET, and SPECT) that are not usually seen by human eyes [69]. As a non-invasive method, it offers several advantages, such as the possibility of studying and following lesions without repeating biopsies [70]. Artificial intelligence (AI), on the other hand, is a branch of computer science encompassing both machine learning and deep learning, and is based on using a training dataset to answer different questions when applied to new data [71]. Using these two techniques in combination with imaging modalities has fundamentally changed the modern era of cancer diagnostics.

Several studies have demonstrated the accuracy and reliability of these techniques in breast cancer diagnosis, staging, prognostication, and treatment response determination [69]. In addition, highly sensitive early screening tests for breast cancer, which by definition have increased cancer detection rates, require follow-up diagnostic biopsies and may introduce unacceptable morbidity to patients with benign or ambiguous lesions. PET or SPECT augmented by AI/radiomics may allow for more accurate lesion description both at the screening and diagnostic phases when compared to conventional methods, circumventing the need for more invasive procedures [72]. However, many of these AI and radiomics models are still in early development phases, and significant external validation and stress testing is needed before they can be safely implemented in routine clinical settings [73].

### 8.3. Multimodality Imaging for Response Assessment

Neoadjuvant (before surgery) application of targeted therapy for breast cancer allows surgeons more flexibility in electing for conservative tumor resections. However, this necessitates highly accurate response assessment by medical imaging in order to allow for rapid application of an alternate therapy or earlier transitions to surgical management if there is failure of the primary treatment [74]. Further complicating this strategy is the fact that there is currently no standardized assessment tool for reporting radiologic response to neoadjuvant therapy, and post-treatment inflammatory changes and calcifications may confound a simple size-based assessment [75].

Currently, mammography, US, and MRI are used for the evaluation of response to neoadjuvant therapy, with 53%, 57%, and 52–61% of patients identified by individual imaging modalities as responders experiencing response on pathology, respectively [76,77,78,79]. However, each modality has specific advantages: ultrasound is more likely to assess early response to therapy while mammography has more potential to detect residual disease prior to surgery, and MRI is the least likely to result in over- or underestimation of remaining tumor size after therapy [74,76]. PET has also shown some promise for evaluating neoadjuvant response and identifying nonresponders, but is overall limited by difficulties in detecting small tumors less than 1 cm [75]. While, in isolation, imaging techniques may have limited accuracy for evaluating the effectiveness of neoadjuvant therapy, further research could focus on a multi-modality approach in which imaging techniques are used in tandem to generate an overall picture of response assessment more closely representative of pathologic findings.

### 8.4. HER2 Intratumoral Heterogeneity

As previously mentioned, even with HER2-targeted therapy, many patients eventually relapse with highly morbid disease. Other than hidden metastatic foci, this can be attributed to intratumoral heterogeneity, in which subpopulations of cancer cells differ genetically and phenotypically—i.e., have differential HER2 expression—from the primary tumor; this occurs in up to 40% of breast cancers [80,81]. When treatment is applied, subpopulations may respond at variable rates, allowing for breakthrough expansion of certain resistant phenotypes. Overall, the presence of HER2 intratumoral heterogeneity is a poor prognostic indicator for those treated with anti-HER2 therapy [82,83]. This represents an ongoing challenge for HER2-targeted diagnosis and treatment, and continuing investigation is necessary to explore the combination of specific and non-specific agents in order to account for molecular pockets of high and low HER2 expression.

### 8.5. HER2 beyond Breast Cancer

Overexpression of HER2 receptors has been reported in many solid tumors other than breast cancer, including gastric cancer, gastroesophageal junction (GEJ) cancer, biliary tract cancer, colorectal cancer, non-small cell lung cancer (NSCLC), and bladder cancer [84].

The incidence of HER2 amplification in gastric cancer is nearly 20%, similar to breast cancer and greater than other solid organ tumors [84]. When compared to breast cancer, HER2 expression in gastric cancer is more heterogeneous, and among different types of gastric cancers, it is more common among the GEJ and intestinal types [85,86]. Furthermore, unlike in breast cancer, in which whole membrane staining is needed to confirm the diagnosis, gastric cancers have basolateral and/or lateral patterns due to their gland-forming nature.

Despite the presence of HER2 overexpression in the aforementioned cancers, the success achieved in treating breast cancers via HER2-targeted therapies cannot be replicated in other solid tumors [84]. Possible reasons for this include increased HER2 receptor heterogeneity as well as genetic diversity in immune cell receptor function, differential density of antigens on tumor cells, and the variable function of immune cells in the setting of each unique tumor environment [87].

## 9. Conclusions

Breast cancer is a widely prevalent cause of morbidity and mortality worldwide, and specific molecular characterization is being explored as an avenue for future precision medicine approaches. Specifically targeting HER2 surface receptors, an EGFR-class protein found in aggressive breast cancers, is an area that is being actively investigated with the aim of offering diagnostic and treatment solutions for certain malignancies. Trastuzumab and pertuzumab are highly effective, antagonist monoclonal antibodies currently used as first-line treatments for HER2-positive breast cancer, and a trastuzumab antibody drug conjugate, T-DXd, was recently approved for use in HER2-low variants (with many others in the pipeline) [88]. Combining these targeted proteins with radionucleotides generates radiotracers that can be visualized by both PET/CT and SPECT/CT imaging for accurate characterization of HER2 presence on both primary and metastatic lesions. Further, with careful selection of the specific tracer used, one could exploit radioactivity to not only provide a visual mapping of disease but also deploy localized radiotherapy in what represents a true diagnostic and therapeutic combined approach. While this latter idea is largely experimental, one could envision a future in which radiolabeled antibody-based scaffolds are used for detailed characterization of an individual patient’s tumor. An imaging signature collected by PET or SPECT imaging could then be processed using AI and radiomics for prognostication as well as prediction of adverse events or treatment response. Lastly, the cancer could be subjected to varying therapeutic approaches, each carefully selected and specific to an individual’s disease. While this summative description of precision medicine may still be theoretical, research in the individual steps is well underway and will soon reach a point when a full connection can be made.

## Figures and Tables

**Table 1:** Summary of in-human studies investigating HER2-targeted radiotracers.

Imaging Modality	n Patients	Radiotracer	Primary Objective	Year	First Author	PMID	Citation
PET/CT	16	^89^Zr-Trastuzumab	Treatment response	2014	Gaykema	25085789	[15]
	11		Detection of HER2+ metastases	2017	Ulaner	28872549	[16]
	14		Feasibility study	2010	Dijkers	20357763	[17]
	9		Detection of HER2+ metastases	2016	Ulaner	27151988	[18]
	20		HER2 status determination	2018	Bensch	30058029	[19]
	34		HER2 status determination	2018	Dehdashti	29442264	[20]
	12		Feasibility study	2016	Laforest	27146421	[21]
	10		HER2 status determination	2019	Jauw	31147401	[22]
PET/CT	22	^89^Zr-Atezolizumab	Feasibility study	2018	Bensch	30478423	[23]
PET/CT	6	^89^Zr-Pertuzumab	Feasibility study	2017	Ulaner	29146695	[24]
	24	^89^Zr-Pertuzumab	Detection of HER2+ metastases	2020	Ulaner	32515679	[25]
PET/CT	1	^89^Zr-Fab *	Feasibility study	2020	Richter, Knorr	32377263	[26]
PET/CT	1	^68^Ga-ZHER2	Detection of HER2+ metastases	2020	Zhou	31833926	[27]
PET/CT	20	^68^Ga-nanobody	Feasibility study	2015	Keyaerts	26449837	[28]
PET/CT	16	^68^Ga-affibody	Feasibility study	2016	Sorensen	26877784	[29]
	24		HER2 status determination	2022	Miao	35712499	[30]
	8		Feasibility study	2016	Sandstrom	26912439	[31]
PET/CT	6	^64^Cu-Trastuzumab	Feasibility study	2013	Tamura	24029656	[32]
	38		HER2 status determination	2017	Sasada	28505219	[33]
	8		Feasibility study	2016	Carrasquillo	27171605	[34]
	5		Detection of HER2+ metastases	2015	Kurihara	25853014	[35]
	8		HER2 status determination	2014	Mortimer	24337604	[36]
	11		HER2 status determination	2018	Mortimer	28637802	[37]
	7		Feasibility study	2021	Lee	33475899	[38]
	1		HER2 status determination	2022	Lee	35133094	[39]
	1		HER2 status determination	2017	Sasada	28770275	[40]
PET/CT	7	^64^Cu-SAR †	Feasibility study	2022	Wong	35890071	[41]
PET/CT	11	^177^Lu-Trastuzumab	Feasibility study	2021	Nautiyal	34406146	[42]
PET/CT	3	^111^In/^68^Ga-affibody	HER2 status determination	2010	Baum	20484419	[43]
SPECT/CT	23		Feasibility study	2017	Sandberg	28261749	[44]
SPECT/CT	7	^111^In-affibody	Feasibility study	2014	Sörensen	24665085	[45]

* Fab indicates antibody fragment. † SAR indicates a Sarcophagine ligand.

## Data Availability

Not applicable.
